# Changes in Heart Rate and Its Regulation by the Autonomic Nervous System Do Not Differ Between Forced and Voluntary Exercise in Mice

**DOI:** 10.3389/fphys.2018.00841

**Published:** 2018-07-16

**Authors:** Robert Lakin, Camilo Guzman, Farzad Izaddoustdar, Nazari Polidovitch, Jack M. Goodman, Peter H. Backx

**Affiliations:** ^1^Department of Exercise Sciences, University of Toronto, Toronto, ON, Canada; ^2^Division of Cardiology, Mount Sinai Hospital, University Health Network, Toronto, ON, Canada; ^3^Department of Biology, York University, Toronto, ON, Canada

**Keywords:** heart rate variability, voluntary exercise, forced exercise, mouse models, cardiac autonomic regulation

## Abstract

Most exercise studies in mice have relied on forced training which can introduce psychological stress. Consequently, the utility of mouse models for understanding exercise-mediated effects in humans, particularly autonomic nervous system (ANS) remodeling, have been challenged. We compared the effects of voluntary free-wheel running vs. non-voluntary swimming on heart function in mice with a focus on the regulation of heart rate (HR) by the ANS. Under conditions where the total excess O_2_ consumption associated with exercise was comparable, the two exercise models led to similar improvements in ventricular function as well as comparable reductions in HR and its control by parasympathetic nervous activity (PNA) and sympathetic nervous activity (SNA), compared to sedentary mice. Both exercise models also increased HR variability (HRV) by similar amounts, independent of HR reductions. In all mice, HRV depended primarily on PNA, with SNA weakly affecting HRV at low frequencies. The differences in both HR and HRV between exercised vs. sedentary mice were eliminated by autonomic blockade, consistent with the similar intrinsic beating rates observed in atria isolated from exercised vs. sedentary mice. In conclusion, both forced and voluntary exercise induce comparable ventricular physiological remodeling as well as HR reductions and HR-independent enhancements of HRV which were both primarily dependent on increased PNA.

**New and noteworthy:**

–No previous mouse studies have compared the effects of forced and voluntary exercise on the heart function and its modulation by the autonomic nervous system (ANS).

–Both voluntary free-wheel running and forced swimming induced similar improvements in ventricular contractile function, reductions in heart rate (HR) and enhancements of HR variability (HRV).

–HR regulation in exercised mice was linked to increased parasympathetic nerve activity and reduced sympathetic nerve activity.

– HRV was independent of HR and depended primarily on PNA in both exercised and sedentary mice.

– Complete cardiac autonomic blockade eliminated differences in both HR and HRV between exercised and sedentary mice.

## Introduction

Studies in mice have been largely limited to forced exercise models, which have been linked to psychological stress (Dishman et al., 2006; Morgan et al., 2015), anti-depressant behavior (Slattery and Cryan, 2012), and elevated corticosterone levels (Gong et al., 2015), though these findings are not universal (Aschar-Sobbi et al., 2015). It has been suggested that psychological stress may limit the physiological remodeling normally seen with exercise training in humans (Billman et al., 2015) in spite of evidence of physiological remodeling in swim models (Evangelista et al., 2003; Aschar-Sobbi et al., 2015). For example, since heart rate (HR) is tightly controlled by the autonomic nervous system (ANS), stress associated with forced exercise can increase sympathetic nerve activity (SNA) limiting the HR reductions typically seen with exercise (Ulrich-Lai and Herman, 2009). Moreover, mice normally display high sympathetic nerve activity (SNA) combined with low parasympathetic nerve activity (PNA) at baseline compared to humans (1996, Gehrmann et al., 2000), suggesting that mice are generally unsuitable for assessing the changes in HR and its regulation by the ANS induced by exercise (Billman et al., 2015). In this regard, although increased PNA and reduced SNA are linked to the HR reductions and increases in HR variability (HRV) induced by exercise (Carter et al., 2003; De Angelis et al., 2004; Billman, 2009), causal links between ANS and HR control have been challenged (Monfredi et al., 2014). In fact, some human studies (Stein et al., 2002; Boyett et al., 2013) as well as a recent study in swim-exercised mice (D'Souza et al., 2014) have concluded that exercise-induced HR slowing (bradycardia) arises exclusively from intrinsic sinoatrial (SA) node remodeling. The origins of HR control and its modulation by exercise are especially relevant because HRV is used to non-invasively assess cardiac autonomic nervous function in the context of heart disease and its treatment by interventions such as exercise (Kishi, 2012; Florea and Cohn, 2014). For these purposes, HRV is dissected typically into rapid “high frequency” (HF) components associated with breathing and PNA as well as slower “low frequency” (LF) components to assess SNA (1996), although this appears to depend on species and experimental conditions (Pomeranz et al., 1985; Pagani et al., 1986).

The effects of exercise on heart function and its regulation have been studied extensively in humans. In particular, it is known that many disease conditions such as heart failure (Scalvini et al., 1998), coronary artery disease (Weber et al., 1999), and hypertension (Konrady et al., 2001) are characterized by lower HRV, and the extent of HRV reduction predicts poor outcomes, including sudden cardiac death (SCD) (Sessa et al., 2018) and responsiveness to therapies (Li et al., 2004). In contrast, increased parasympathetic tone and sympathetic withdrawal have been linked to cardioprotection and improved outcomes in heart failure (Imai et al., 1994) and cardiovascular disease (Laurita and Hirose, 2013), which may underlie many of the positive effects of exercise therapy in heart disease patients (Coats et al., 1992).

In our study, we compared the effects of voluntary free-wheel exercise and forced swim exercise on ventricular structure and function as well as the changes and regulation of both HR and HRV by the ANS.

## Methods

### Experimental animals and endurance exercise protocols

This study was carried out in accordance with the recommendations of the Canadian Council of Animal Care. The protocol was approved by the Division of Comparative Medicine at the University of Toronto and York University Animal Care Committee. 6-week-old CD1 male mice (body weight = 28–34 g, Charles River Laboratories) were housed in a room with a constant temperature (22 ± 1°C) with a 12-h:12-h light-dark cycle and fed a standard laboratory mouse diet *ad libitum* with free access to water. Mice were randomly assigned to sedentary (*n* = 22) or 6-week swimming (*n* = 32) or free-wheel running (*n* = 22) groups. For 6-week swim exercised mice, mice swam during the light phase of their light-dark cycle in swim containers (30 cm diameter) at 33°C (Supplementary Video 1). To avoid drowning stress associated with the use of tail-weights, bubbling, or detergents, swim exercised mice swam in tanks equipped with pumps to generate steady currents (10–20 l•min^−1^), which we previously showed does not cause plasma elevations in stress hormones (Aschar-Sobbi et al., 2015). Swim training began with 30-min swim sessions (twice daily, separated by 4 h) that were increased to 90-min by 10-min increments per day followed by 6 weeks of training. For free-wheel running, cages were equipped with unlocked (non-resistance) free-wheels which mice were allowed to voluntarily use over the course of 6 weeks. Speed and daily wheel revolutions were recorded digitally using custom software running on Raspberry Pi computers and distance was calculated by the formula:

$$\begin{array}{l} \text{Distance }\left(\text{km}\right)\;=\text{ Wheel Revolutions }*\;\left(28.8\text{cm}/100,000\right) \end{array}$$

Sedentary mice were placed in swim containers without a water current for 5-min each swim session to ensure similar handling, or placed in cages in which the free-wheels were locked in an immobile position for the duration of the experiment. For morphological measurements, mice were euthanized and whole hearts and individual heart chambers were weighed (Table 1).

**Table 1:** Physical characteristics of mice.

	**Sedentary**	**Swim**	**Free-wheel Running**
**Body parameters** ***n***	**13**	**14**	**11**
Body weight (g)	41.5 ± 2.5	36.3 ± 3.7^*^	35.7 ± 3.0^*^
Tibia length (mm)	19.3 ± 0.7	19.1 ± 0.7	19.2 ± 1.0
Atria weight (mg)	17.7 ± 2.2	19.2 ± 1.5	18.5 ± 2.0
Ventricle weight (mg)	156.2 ± 17.3	145.7 ± 8.2	152.0 ± 12.6
Atria/tibia length (mg mm^−1^)	0.90 ± 0.11	0.99 ± 0.07^*^	0.96 ± 0.13
Ventricle/tibia length (mg mm^−1^)	8.09 ± 0.94	7.67 ± 0.41	7.92 ± 0.60
Atria/body weight (mg g^−1^)	0.43 ± 0.07	0.54 ± 0.07^*^	0.52 ± 0.10^*^
Ventricle/body weight (mg g^−1^)	3.76 ± 0.29	4.06 ± 0.27^*^	4.26 ± 0.33^*^
Atria/ ventricle weight (mg g^−1^)	114.4 ± 18.4	132.2 ± 13.5^*^	121.7 ± 12.6

*The n value shown represents the number of mice used. Data presented as mean ± SD*.

* *P < 0.05, compared with sedentary using Student's t-test corrected for multiple comparisons*.

### Echocardiography

Left ventricular functional and morphological remodeling was assessed as previously described (Aschar-Sobbi et al., 2015). Briefly, mice were anesthetized with 1.5% isoflurane oxygen mixture. Mice were placed on a heating pad and body temperature was maintained between 36.9 and 37.3°C for the duration of the measurements. Transthoracic M-mode echocardiographic examination was conducted using a Vevo 2100 system (VisualSonics) equipped with ultrasonic linear transducer scanning heads operating at 30 MHz. The left ventricular long-axis view was used for measurement of functional indices, chamber size, and wall diameter. Data analysis was performed using the VisualSonics data analysis suite.

### Electrocardiography

Surface ECG measurements were undertaken on mice anesthetized using a 1.5% isoflurane oxygen mixture with sub-dermal platinum electrodes placed in lead II arrangement. Internal body temperature was maintained between 36.9 and 37.3°C. All data were analyzed using Ponemah Physiology Platform (P3) software. To assess parasympathetic and sympathetic tone, HRs of anesthetized mice were monitored at baseline and following sequential pharmacological blockade with intraperitoneal injections of atropine sulfate (2 mg•kg^−1^ BW) and/or propranolol hydrochloride (10 mg•kg^−1^ BW) (Sigma-Aldrich) to block the parasympathetic and sympathetic branches of the autonomic nervous system, respectively.

### HR and HR variability (HRV) analysis in anesthetized mice

The bulk of our studies used surface ECG recordings in anesthetized mice after completing their 6 weeks of exercise to assess the effects of exercise on HR, HRV and autonomic nervous system (ANS) remodeling. All measurements were made 72 h after the last exercise bout to avoid the possible confounding influence of acute exercise on HRV and cardiac ANS measurements (Valenzano et al., 2016). For these measurements, a minimal depth of anesthesia was strictly maintained at equivalent levels between sedentary and exercised mice as determined by a breathing rate of 90–110 breaths per minute combined with the loss of the “toe pinch-pedal reflex.” Surface ECG recordings were marked for R-R intervals using P3 Plus (Data Sciences International), and the raw text files of inter-beat-intervals (IBIs) were imported to Kubios HRV software (University of Eastern Finland) for analysis. Mean HRs were estimated by averaging the inverse of the RR intervals. HRV was analyzed by performing Fast Fourier Transforms (FFTs) on the contiguous R-R intervals recorded over a period lasting a minimum of 5 min. The FFT generated complex amplitudes as functions of frequency. The spectral power density of the HRV was calculated by taking the complex conjugate of the amplitudes of the FFT for each frequency. The total spectral power (TP) of the HRV was estimated by integrating the HRV spectral power from frequencies between 0.1 and 5 Hz. Integration of the HRV spectral power from 0.1 to 1 Hz was used to estimate the low frequency (LF) HRV and from 1–5 Hz to estimate the high frequency (HF) HRV, respectively, which we determined previously using atropine (2 mg kg^−1^) and propranolol (10 mg kg^−1^)(Aschar-Sobbi et al., 2015). To assess the role of parasympathetic and sympathetic nervous systems in HRV, mice were treated with atropine (2 mg•kg^−1^) and propranolol (10 mg•kg^−1^), respectively, which resulted in complete cardiac autonomic blockade, as we have shown previously (Aschar-Sobbi et al., 2015). Power spectral density of the HRV was determined from FFTs of the ECG recordings using Welch's periodogram at an interpolation rate of 20 Hz, with a 256 s window and 50% overlap of windowed segments (Tarvainen et al., 2014). Because HRV appears to decrease intrinsically as HR increases (Sacha and Pluta, 2008), independently of cardiac autonomic nerve activity, we also corrected our HRV for HR differences between the groups. This was done by multiplying the TP, LF, and HF components of the HRV by the mean HR squared (i.e., HR^2^ [Hz]) for each animal, as done previously (Billman, 2013).

### HR and HR variability (HRV) analysis in conscious mice

To assess the effects of exercise on HR and HRV in conscious mice, we inserted ECG telemetry devices (ETA-F10, Data Sciences International) into the abdominal cavity. Briefly, the negative lead was tunneled subcutaneously from the thorax to the neck and fixed between the muscles located to the right of the trachea. The positive electrode was sutured to the xiphoid process such that it was located between the liver and the diaphragm in the left upper abdominal region. After 10 days of recovery, mice underwent the swim training protocol. Data analysis on conscious, telemetry-implanted mice was processed using DataQuest A.R.T. Analysis software (Data Sciences International) and ECG recordings were exported to Kubios HRV software (University of Eastern Finland) and analyzed by performing FFTs, as described above.

### Isolated denervated atrial recordings

Heparinized mice were anesthetized using isoflurane and sacrificed via cervical dislocation. The thorax was opened by midsternal incision, and the heart was excised into warm (35°C) Tyrode's solution (in mmol/l): 140 NaCl, 5.4 KCl, 1.2 KH_2_PO_4_, 1 MgCl_2_, 1.8 CaCl_2_, 5.55 D-glucose, 5 HEPES, and 10 U•ml^−1^ heparin (pH 7.4). The heart was pinned to a Sylgard-coated petri dish with insect pins to reveal the dorsal atrioventricular connective tissue. The pericardium and other residual lung and connective tissue were carefully excised from the heart. A small lining of adipose tissue guided incisions along the atrioventricular connective tissue until complete separation of the atria from the ventricles was achieved. Atria were then pinned so as to reveal the mitral and tricuspid valves. Fine scissors were inserted into the superior and inferior vena cava, and incisions were made in a straight path to “open” the atria. The atria were then turned to reveal the pulmonary veins, and residual lung tissue was removed. Atria were then transferred and continuously superfused using 35°C carbogenized (95% O_2_ and 5% CO_2_) Krebs solution (in mmol l^−1^): 118 NaCl, 4.2 KCl, 1.2 KH_2_PO_4_, 1.5 CaCl_2_, 1.2 MgSO_4_, 2.3 NaHCO_3_, 20 D-glucose, 2 Na-pyruvate (pH measured at 7.35–7.4). The beating rates of isolated, denervated atria were then determined using a 3-lead ECG set up in the Sylgard dish and analyzed using AxoScope (Axon Instruments, CA, USA).

### VO_2_ consumption measurements

Oyxgen consumption rates (VO_2_) were measured during rest (sleep periods), ambulatory activity (awake periods), and during exercise by indirect calorimetry using the Comprehensive Lab Animal Monitoring System (CLAMS) or the Oxymax oxygen monitoring system (Columbus instruments, Columbus, OH). For swimming, mice were placed in a sealed container containing a water pump to generate a current (33°C). The maximum oxygen consumption (VO_2max_) rates were estimated from the plateau in VO_2_ observed during swimming. For free-wheel running, mice were placed into a modified CLAMS cage equipped with a free-wheel and VO_2_ was measured during running activity detected via electronic sensors on the wheel and correlated with maximal running speed (cm/second). All VO_2_ data was normalized to body weight.

### Corticosterone measurements

A subset of mice (*n* = 7 per group) were pre-selected from non-exercised control, swim, and free-wheel exercised mice. The exercised groups were exercised according to the protocols described above for 1 week (after the initial familiarization phase) prior to fecal sample collection. To avoid acute effects, fecal samples were collected within 15–16 h following the final exercise session, at the same time of day in all groups (during light cycle, 8:00–9:00 a.m.). Samples were homogenized and dissolved in methanol (1 ml/0.05 g feces). Samples were vortexed for 30 min, followed by centrifugation at 4,000 x g for 10 min. Samples were then diluted 1:10 using assay buffer provided by the manufacturer (Enzo Life Sciences ELISA kit). The corticosterone was measured according to manufacturer instructions.

### Statistical analysis

Data are presented as mean (standard deviation). Normality of data distribution was examined using a Kolmogorov-Smirnov test. In case non-Gaussian distribution was observed, log-transformation was applied after which the data was re-examined. Statistical significance was determined using unpaired or paired student's *t*-test (two-tailed) as appropriate, a one-way ANOVA with Sidak's multiple comparison test, or a two-way ANOVA with Sidak's multiple comparison test. A *P*-value of < 0.05 was considered significant. Welch's *t*-test was used when the variance differed between groups (*F*-test). Outliers were identified using Grubbs' test, also known as the ESD method (extreme studentized deviate), and removed where necessary. When no differences (*P* > 0.05) between the forced and voluntary exercise groups were observed (as was often seen), and when differences (*P* < 0.05) between the combined exercise groups vs. the sedentary mice were observed, we report an exercise group *P*-value. All data were analyzed using GraphPad 6.04 software (GraphPad Software Inc., San Diego, CA).

## Results

### O_2_ consumption and cardiac function in exercised mice

VO_2_ were measured during exercise in order to assess the levels of effort and work performed associated with exercise over the 6-week training period. VO_2_ during swimming were 11,260 ± 563 vs. 5,534 ± 448 mls O_2_/kg/h (*P* < 0.0001) when voluntary running on the free wheels during dark periods. By contrast, the VO_2_ during awake periods when mice were ambulatory but not exercising were 3,033 ± 192 mls O_2_/kg/h in the swim group and 3,117 ± 254 mls O_2_/kg/h in the free-wheel group, which was similar (*P* = 0.748) to that recorded in sedentary mice (3,019 ± 200 mls O_2_/kg/h). Despite differences in VO_2_ during exercise, the estimated integrated excess O_2_ consumption associated with exercise (above baseline) over the 6-week period of exercise were similar between the two exercise groups. Specifically, with free-wheel exercise mice ran for 5.13 ± 0.44 h/day and consumed an estimated total O_2_ (above baseline) of 1,192 ± 328 l/kg in association with exercise over 6 weeks which was not different (*P* = 0.407) from the total O_2_ 1,013 ± 319 l/kg (above baseline) associated with exercise when mice swam for 180 min/day over the same 6 week period. These results demonstrate that the amount of work performed during exercise over the 6 week period was similar between the groups.

Consistent with the VO_2_ measurements, the two exercise groups showed similar (*P* > 0.109) responses to exercise compared to sedentary mice, characterized by reduced (*P* < 0.0007) HRs, reduced (*P* < 0.0003) body weights, increased (*P* < 0.010) ventricular weights (normalized to body weight), and increased (*P* < 0.0001) LV end-diastolic diameters (Tables 1, 2). Moreover, contractility, as assessed by fractional shortening, was reduced (*P* < 0.041) at rest, consistent with chamber dilatation. The similar degree of cardiac remodeling seen in forced swim exercise mice compared to voluntary free-wheel running mice suggests that the potential stress associated with forced swimming during the light (sleeping periods) does not compromise the normal physiological remodeling associated with exercise (see section Discussion). In agreement with this suggestion, fecal corticosterone levels (ng/0.05 g feces) being similar (*P* = 0.118) between free-wheel (9.54 ± 1.72), swim exercised (8.34 ± 1.96), and sedentary (7.51 ± 1.51) mice.

**Table 2:** Left ventricular echocardiographic parameters.

	**Sedentary**	**Swim**	**Free-wheel Running**
**Echocardiography** ***n***	**22**	**32**	**22**
LVDd (mm)	4.24 ± 0.13	4.48 ± 0.18^*^	4.50 ± 0.09^*^
LVDs (mm)	2.70 ± 0.19	2.93 ± 0.24^*^	3.01 ± 0.28^*^
SV (μl)	53.4 ± 3.8	56.0 ± 6.0	56.4 ± 9.4
EF (%)	67.0 ± 4.4	63.0 ± 5.4^*^	61.5 ± 8.9^*^
FS (%)	36.3 ± 3.2	34.6 ± 3.2^*^	33.1 ± 3.6^*^
CO (ml min^−1^)	27.3 ± 3.8	25.0 ± 4.8	26.0 ± 8.9
HR (bpm)	511 ± 57	448 ± 72^*^	456 ± 89^*^
LVPWth (mm)	0.79 ± 0.06	0.76 ± 0.06^*^	0.79 ± 0.05

*CO, cardiac output; EF, ejection fraction; FS, fractional shortening; LVDd, left ventricular diastolic diameter; LVDs, left ventricular systolic diameter; LVPWth, left ventricular posterior wall thickness. The n value shown represents the number of mice used. Data presented as mean ± SD*.

* *P < 0.05, compared with sedentary using Student's t-test corrected for multiple comparisons*.

### The effects of exercise on HR and its control by the ANS

**Figure 1:**
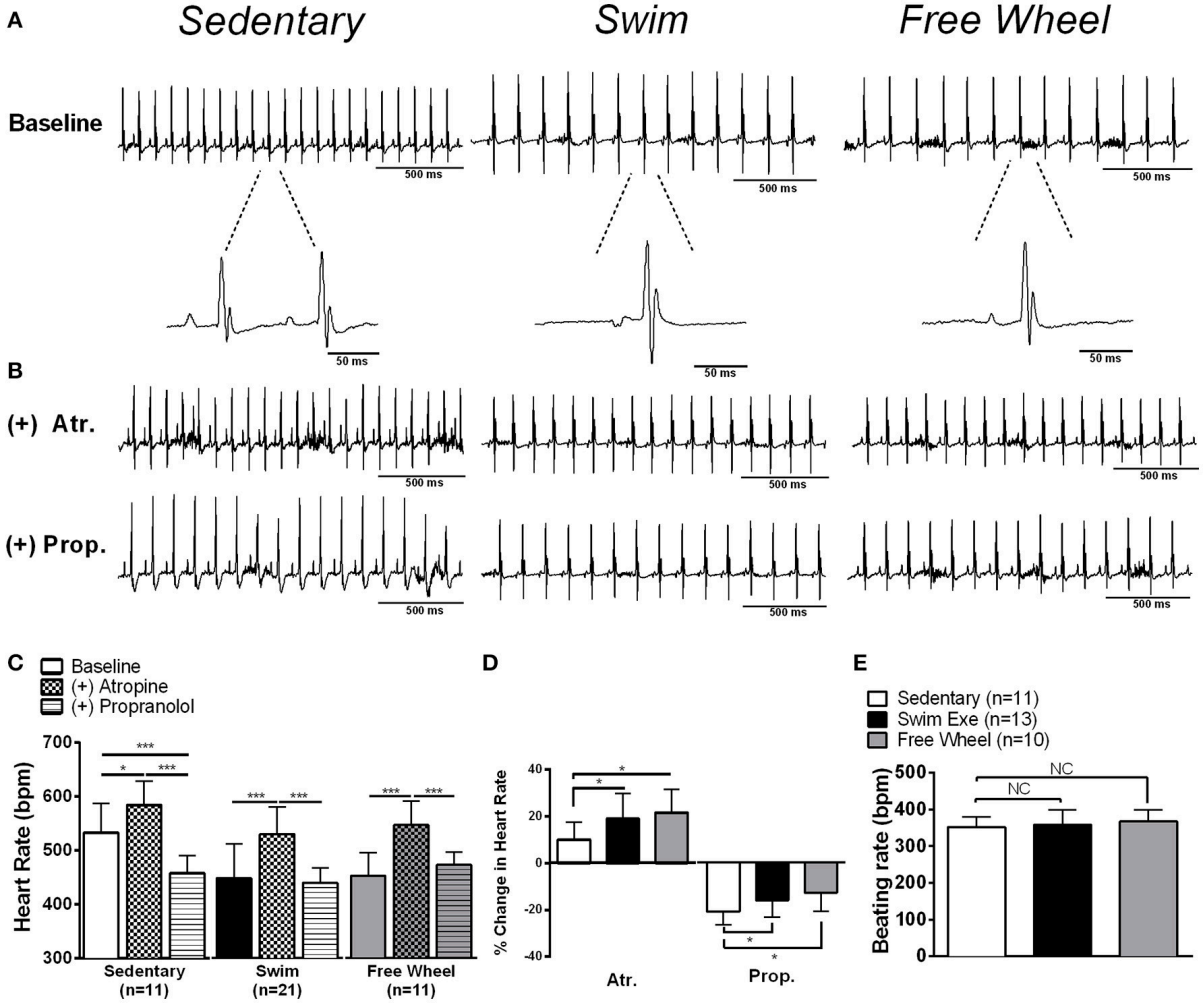
Exercise induces sinus bradycardia modulated by the autonomic nervous system in mice.**(A)** Representative surface ECG recordings of sedentary, swim, and free-wheel exercised mice showing reduced heart rates (HRs) in exercise-trained mice. **(B,C)** Pharmacological blockade of PNA alone increased HR, while blockade of SNA reduced HR in all groups. **(D)** The percentage change in HR induced by PNA or SNA blockade. Exercised mice had a higher percentage change in HR with PNA blockade (*P* < 0.021) and a lower percentage change with SNA blockade (*P* < 0.044), suggesting a shift toward increased PNA and lower SNA with exercise. **(E)** Beating rates in isolated denervated atria isolated from swim exercised, free-wheel exercised and sedentary mice revealed no significant (*P* = 0.61) differences between groups. Data is presented as mean±SD. ^*^P < 0.05*;* ^***^P < 0.001.

To examine whether the HR reductions (Table 2) with exercise were associated with ANS remodeling, surface ECGs were recorded in anesthetized mice in the presence and absence of pharmacological blockers of SNA (propranolol hydrochloride, 10 mg•kg^−1^), PNA (atropine sulfate, 2 mg•kg^−1^), or both. Although our analyses compared HRs between the groups, our conclusions were the same when R-R intervals were compared. Consistent with the echocardiographic recordings (Table 2), Figure 1 shows that baseline HRs were lower (*P* < 0.001) by similar amounts in swim (449 ± 64 bpm) and free-wheel (453 ± 43 bpm) exercised mice, compared to sedentary mice (533 ± 53 bpm). Following PNA blockade, HR increased in sedentary (*P* = 0.024) and both exercise groups (*P* < 0.001), with the relative HR increase being greater (*P* < 0.05) in both swim- and free wheel-exercised mice compared to sedentary mice (Figure 1D). Subsequent SNA blockade with propranolol, in the continued presence of atropine, led to reductions (*P* < 0.001) in HR in all three groups (Figure 1C) with the relative HR reduction being less (~25%)(*P* < 0.040) in exercised than sedentary mice (Figure 1D). After complete autonomic blockade (i.e., both atropine and propranolol), HRs did not differ (*P* > 0.210) between exercised and sedentary mice. This interpretation is further supported by the observation (Figure 1E) that the beating rates in isolated (denervated) hearts did not differ (*P* = 0.61) between groups (sedentary: 352 ± 27, swim: 359 ± 41, free-wheel: 368 ± 32).

**Figure 2:**
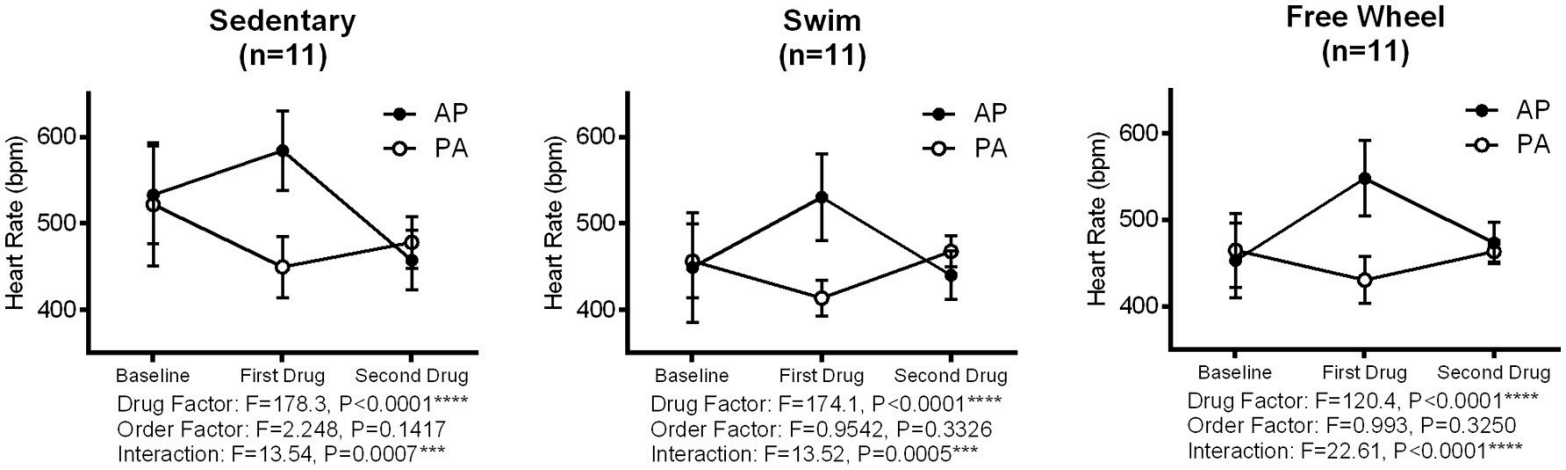
Influence of sequential administration of atropine and propranolol, and drug order effects, on heart changes in sedentary and exercised mice. Representative changes in HR following sequential administration of atropine (A) (2 mg/kg) and propranolol (P) (10 mg/kg) in two opposite orders (i.e., AP and PA) are shown. Two-way ANOVA indicated a significant main effect for drug (*P* < 0.0001, all groups) and interaction between drug and order (*P* < 0.0007 in all groups), with no effect of order observed (*P* > 0.14, all groups). In all groups, atropine had a larger effect (i.e., 1.5-to-2-fold) (*P* < 0.0001) on HR increase when administered in the absence compared to the presence of propranolol. In contrast, propranolol had a larger effect (i.e., 1.5-to-2-fold) (*P* < 0.0001) on HR when administered after atropine. These results are indicative of “accentuated antagonism.” Solid circles (•) indicate the atropine-propranolol (AP) sequence, while open circles (°) indicate the propranolol-atropine (PA) sequence. Data are given as means ± *SD*.

Although our results might suggest that the reduced HR induced by both forced and voluntary exercise arises from increased PNA, and reduced SNA, this interpretation is complicated by potential (feedback) interactions between the PNA and SNA. Therefore, we also reversed the order of autonomic blockade (Figure 2). Consistent with feedback interactions, two-way ANOVA revealed a dependence (*P* < 0.0007) on the order of drug administration in all three groups. This interaction can be readily appreciated by noting that there was a 1.5-to-2-fold greater (*P* < 0.0001) HR increase when atropine was administrated in the absence of propranolol vs. when given in the presence of propranolol in all groups. Moreover, we found a 1.5-to-2-fold greater (*P* < 0.001) decrease in HR by propranolol in the presence vs. the absence of atropine in all groups. On the other hand, consistent with Figure 1, the HR increases (*P* < 0.001) with PNA blockade were greater and the HR reductions (*P* < 0.01) with SNA blockade were blunted in both exercise groups compared to sedentary mice, regardless of the order of drug administration (*P* > 0.239).

### The effects of exercise on HRV and its regulation by the ANS

**Figure 3:**
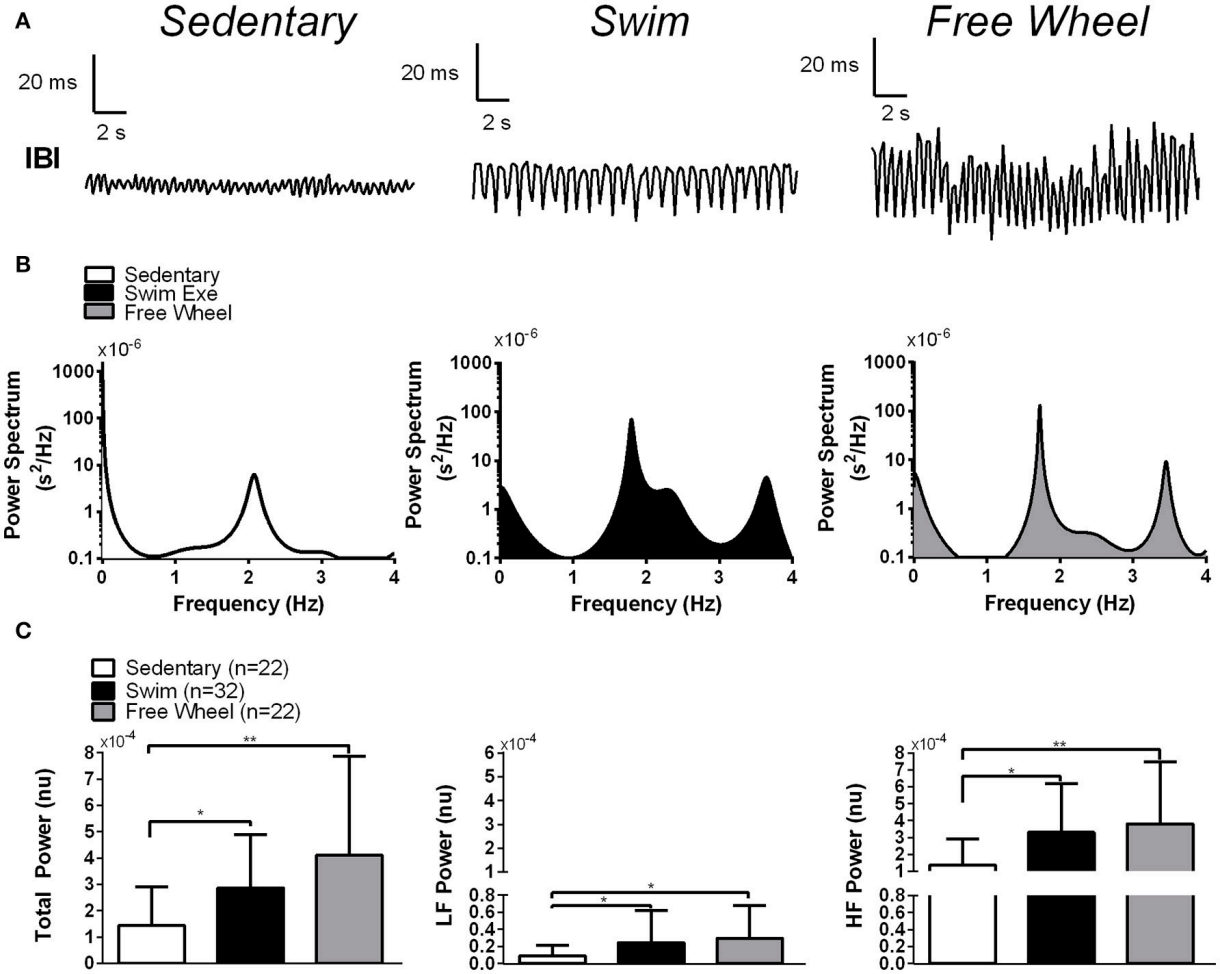
Effects of exercise and cardiac autonomic blockade on HRV. **(A)** Representative inter-beat-intervals (IBIs) as a function of time in a swim exercised, a free-wheel exercised and a sedentary mouse. The results clearly show increased fluctuations of the R-R intervals in exercise-trained mice compared to sedentary controls. **(B)** Typical examples of spectral power of the HRV (generated by Fast Fourier transforming the R-R intervals) (1996) as a function of frequency for a swim exercised mouse, a free-wheel exercised mouse and a sedentary mouse. Notice the clear increase in the amplitude of the spectral power in the exercised mice compared to the sedentary mouse at virtually all frequencies. **(C)** Integration of the spectral density of the HRV from 0.1 to 5.0 Hz (Total Power, left panel), from 0.1 to 1.0 Hz (Low Frequency Power, middle panel) and from 1.0 to 5.0 Hz (High Frequency Power, right panel) in sedentary, swim exercised and free-wheel exercised mice, corrected for HR (by multiplying by HR^2^, see section Methods). In our analysis, three outliers were identified using Grubb's test (*n* = 1 for the swim group) and (*n* = 2 for free-wheel group) which had HRV well beyond the upper limit of our data distribution. Power spectral data is presented as mean±*SD*. ^*^P < 0.05*;* ^**^P < 0.01.

To assess HRV changes with exercise we performed Fourier transforms of the inter-beat-intervals (IBIs) on sequences of R-R intervals (Figure 3A). Crude inspection of the results in Figure 3B suggests that both exercise groups possess greater spectral densities, and therefore greater HRV, compared to sedentary mice. To quantify the HRV further, the HRV spectral density was integrated over several frequency ranges (see section Methods). The total power (TP) of the HRV was calculated as the sum of the LF and HF components. Since the magnitude HRV depends on HR, irrespective of autonomic activity changes (Monfredi et al., 2014), we corrected for differences in basal HR between the groups by multiplying HRV by the HR^2^ (see section Methods). Figure 3C shows that LF, HF, and TP were not different (*P* > 0.171) between swimming and free-wheel running mice but were elevated (*P* < 0.038) in the exercised groups compared with sedentary mice. Unless stated otherwise, all subsequent HRV results were corrected for HR.

**Figure 4:**
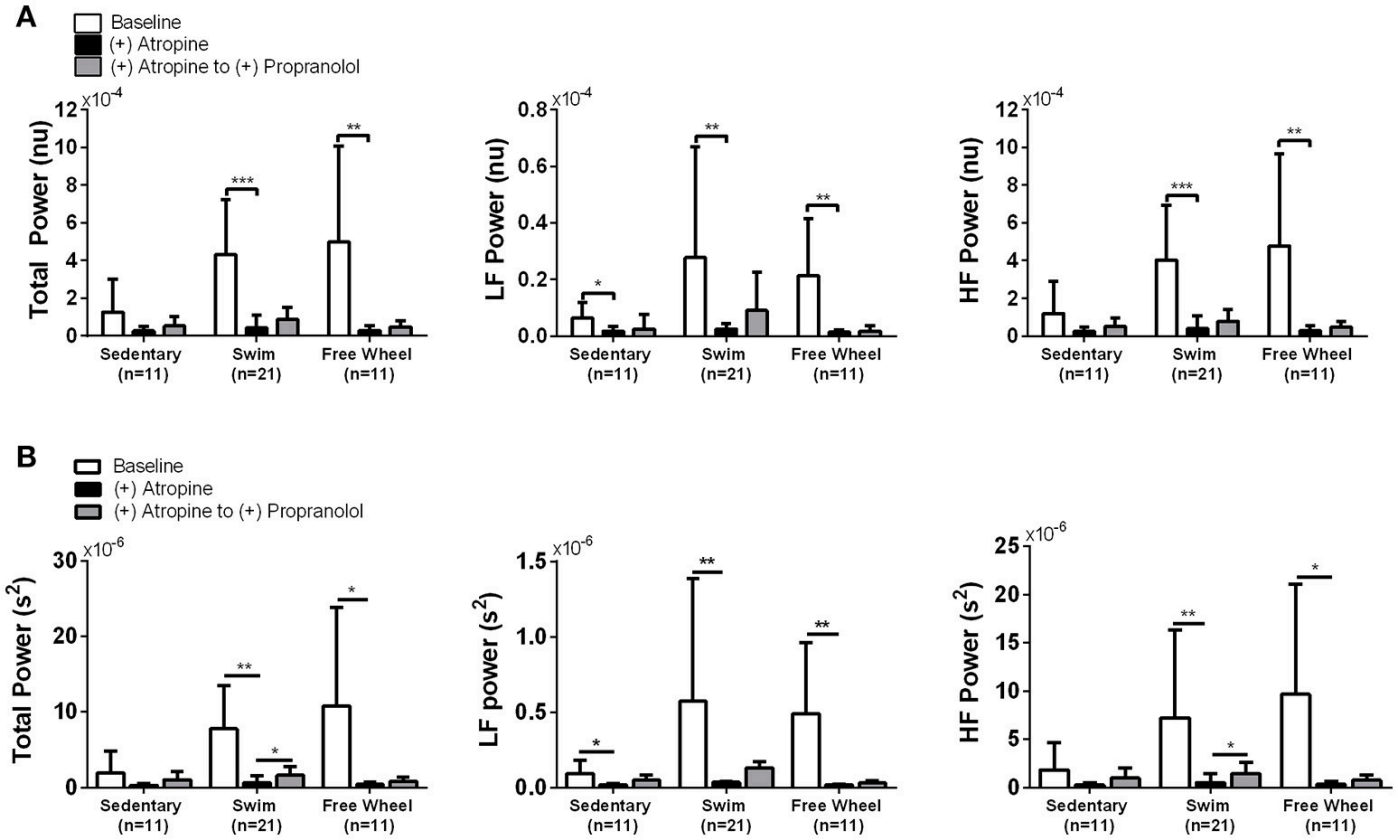
Effects of cardiac autonomic blockade on HRV. **(A)** HR corrected total, LF, and HF power of the HRV in the absence of cardiac autonomic blockade (Baseline), after atropine, and after atropine plus propranolol. Total, LF, and HF power of the HRV were higher in exercised mice compared to sedentary mice. Atropine treatment reduced total (*P* < 0.006), LF (*P* < 0.008), and HF (*P* < 0.006) power of the HRV in exercised mice. Atropine also reduced LF HRV (*P* = 0.011) with evidence of reductions in TP (*P* = 0.075) and HF (*P* = 0.081) HRV in sedentary mice that did not reach significance. Following propranolol administration in the presence of atropine, there was a mean increase (*P* = 0.051) in TP and HF HRV for swim exercised mice. **(B)** The same data as in **(A)** but in the absence of HR corrected HRV. The differences in TP, LF, and HF HRV identified between the groups of mice are unchanged without HR correction, suggesting HR has a minimal effect on HRV in the current study. Data are presented as mean ± *SD*. ^*^P < 0.05; ^**^P < 0.01; ^***^P < 0.001.

**Figure 5:**
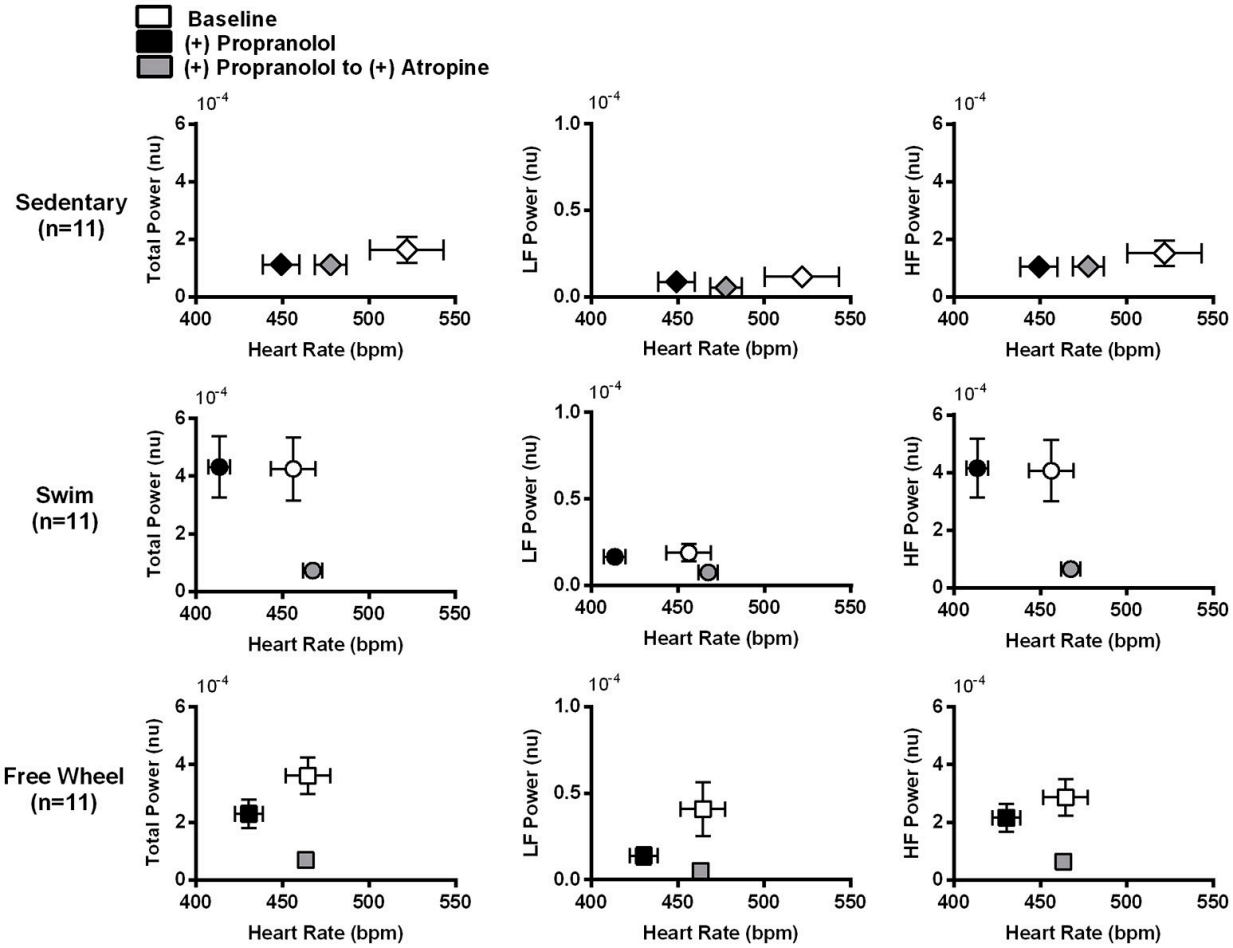
Change in HRV as a function of HR induced by SNA blockade followed by PNA blockade. Plots of the TP, LF, and HF HRV, corrected for HR, as a function of HR at baseline (no drug), after propranolol, and after propranolol plus atropine. Propranolol administration reduced HR without affecting TP, LF, or HF HRV in exercised as well as sedentary mice. The subsequent administration of atropine, in the presence of propranolol, reduced TP (*P* < 0.005) and HF (*P* < 0.007) HRV while increasing (*P* < 0.001) HR in both exercise groups, similar to the responses to atropine in the absence of SNA blockade. Atropine had no measurable (*P* = 0.961) effect on HRV in sedentary mice despite also increasing HR. After complete cardiac autonomic blockade (propranolol + atropine), HR differences between groups were eliminated (*P* > 0.301). Data are presented as mean ± SD.

Figure 4A reveals that PNA blockade reduced TP (*P* < 0.006) and HF power (*P* < 0.006) of the HRV in swim and free-wheel exercised mice compared to baseline before drug. Atropine also reduced the LF power of the HRV in swimming (*P* = 0.008), free-wheel running (*P* = 0.004), and sedentary (*P* = 0.011) mice. While the influence of PNA blockade on the HF HRV is expected (Pomeranz et al., 1985), the effects of PNA blockade on LF HRV is somewhat surprising since LF HRV has been used in humans as a barometer of SNA (Pomeranz et al., 1985; Pagani et al., 1986), although this observation is consistent with previous human (Pomeranz et al., 1985; Grasso et al., 1997) and mouse (Gehrmann et al., 2000) studies. Moreover, HRV responses to PNA blockade were also similar without HR corrections (Figure 4B). Consistent with the HR results, the differences in HRV between the exercised and sedentary mice were eliminated by PNA blockade, supporting the conclusion that exercise-induced HRV elevations depend on increased HR control by the PNA. By contrast, blockade of sympathetic activity with propranolol in the continued presence of atropine (as well as in the absence of atropine, see Figure 5 below) had minimal impact on HRV for all groups (Figures 4A,B). Unexpectedly, despite evidence of a ~25% reduction of baseline HR control by SNA in exercised mice, propranolol administration after atropine treatment tended to increase the HRV in all groups.

**Figure 6:**
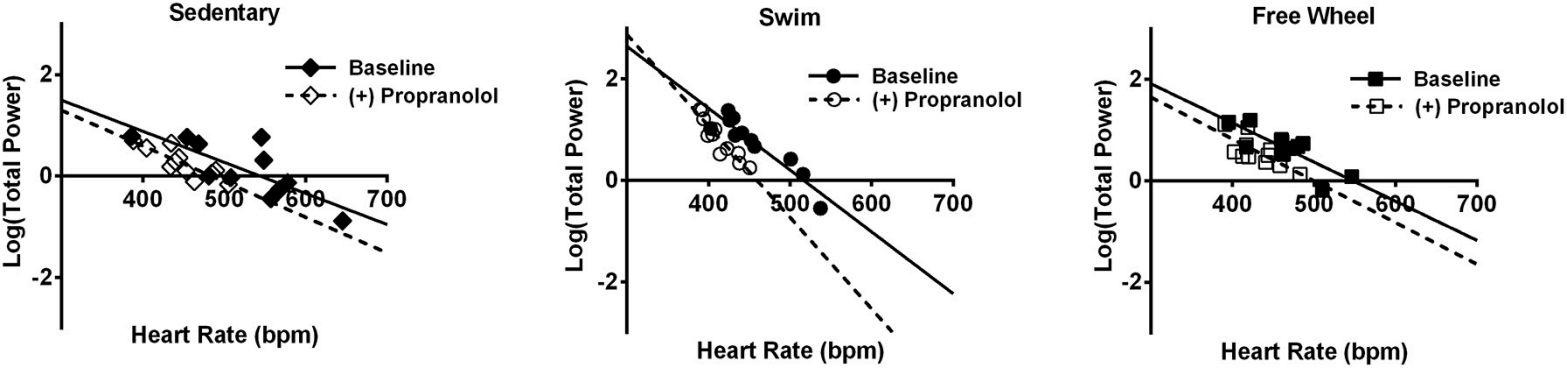
Relationship between TP HRV and HR before and after propranolol. In the absence of propranolol, linear regression analyses of the relationship between the logarithm of the TP HRV and the HR, revealed that HRV decreased exponentially for both sedentary and exercised mice with the dependence being somewhat steeper (*P* < 0.054) for the swim exercised mice (*n* = 11, slope = −0.012 ± 0.0016, *r* = 0.93) vs. free-wheel (*n* = 11, slope = −0.0077 ± 0.0018, *r* = 0.82) and sedentary mice (*n* = 11, slope = −0.0061 ± 0.0016, *r* = 0.78). Propranolol administration caused an increased, albeit non-significant (P = 0.054), dependence of TP HRV on HR in swim exercised mice (slope = −0.018 ± 0.0024, *r* = 0.93), driven by a reduction in the range of HR values following SNA blockade. No change in the relationship was observed in free-wheel (*P* = 0.86) or sedentary (*P* = 0.68) mice following propranolol administration showing that HRV is not highly dependent on HR independently of PNA. No change (*P* > 0.12) in the intercept was observed in any group. The nature of the relationship did not change in the presence or absence of HR correction (data not shown).

To ensure that our conclusions regarding the changes in HRV and its control by the ANS induced by exercise are independent of feedback interactions between PNA and SNA, we also analyzed HRV changes when the order of autonomic blockade was reversed. For these findings we chose to present our results in a different format thereby highlighting further that the exercise-induced increases in HRV are not HR-dependent, as suggested previously (Gehrmann et al., 2000; Monfredi et al., 2014). Indeed, even though propranolol reduced HR (*P* < 0.046) in all three groups of mice, it had no measurable effects on HRV and, therefore, the exercise-induced increases in HRV remained (*P* < 0.048) after SNA blockade (Figure 5). This conclusion is further illustrated in Figure 6 which plots the TP of the HRV as a function of HR on a logarithmic scale. Consistent with previous studies demonstrating a dependence of HRV on HR (Monfredi et al., 2014), the logarithmic relationship between the TP of the HRV and HR is linear, with steeper (*P* < 0.054) dependence for the swim group compared to either the free-wheel or sedentary groups. More important, propranolol shifted the relationship between HRV and HR leftward without affecting the slope or the magnitude of the HRV. By contrast, blockade of PNA, in the presence of propranolol, reduced both TP (*P* < 0.005) and HF (*P* < 0.007) HRV, while having no measurable (*P* = 0.961) effects on HRV for sedentary mice. Again, complete cardiac autonomic blockade eliminated HR differences between groups.

## Discussion

Our results are the first to comprehensively demonstrate that voluntary free-wheel running and forced swimming induce similar changes (HR, heart structure, and function) that mirror the “athlete's heart” in humans. It has previously been suggested that forced exercise, which is commonly used to model exercise in mice, may introduce aversive (i.e., drowning stress, electrical shock) stimuli thereby limiting the ability to fully recapitulate the athlete's heart phenotype (Billman et al., 2015). Despite the possibility that the psychological stress associated with forced training might mask or prevent exercise-induced physiological remodeling (Bartolomucci et al., 2003), we found that swimming induced similar structural, functional, and autonomic changes as seen with voluntary free-wheel running. Moreover, the HR reductions and increased HRV with both types of training were eliminated by cardiac autonomic blockade and were absent in isolated atria, suggesting that the changes in HR and HRV are dependent on ANS remodeling. Indeed, both exercise groups showed increases in HR control by PNA (~2-fold) and decreases by SNA (~25%). The similar responses seen between the groups are even more surprising given that mice swam twice per day during the light period (of the 12-h:12-h light-dark cycle), which required sleep interruption, and that forced swimming involved training intensities during swimming (as measured by O_2_ consumption rates) that were more than 2-fold greater than during free wheel running. Thus, despite the stress associated with our forced swimming protocols, beneficial physiological remodeling was not impeded.

The similar cardiac changes seen with forced and voluntary exercise in mice was associated with similar fecal corticosterone levels between groups, suggesting that the impact of stress associated with forced swimming does not compromise the beneficial effects of exercise. However, interpretation of corticosterone measurements can be complicated (Skoluda et al., 2012). Specifically, sleep interruption and/or deprivation associated with our forced exercised protocol might have flattened the circadian pattern of corticosterone levels (Dmitrieva et al., 2013; Desantis et al., 2015). Moreover, although fecal corticosterone levels were measured at the beginning of the light cycle (8:00–9:00 a.m.), which is typically associated with high levels of stress hormones in rodents (Cinque et al., 2018) and humans (Kirschbaum and Hellhammer, 1989), it is possible that corticosterone levels may have differed at other time points. It is also possible that the effects of stress associated with forced exercise could unfold if the mice were exercised for longer periods of time.

Our findings that the changes in HR induced by exercise are mediated by ANS remodeling are consistent with previous mouse (De Angelis et al., 2004), dog (Ordway et al., 1982; Billman et al., 2015), and human (Shi et al., 1995; Carter et al., 2003) studies. However, a previous mouse study concluded that the bradycardia induced by swim exercise arises exclusively from intrinsic ion channel remodeling in the sinoatrial node (D'Souza et al., 2014), consistent with some human studies (Boyett et al., 2013). The basis for the lack of agreement between our study and that of D'Souza et al. (2014) is unclear but may be related to differences in the swimming duration (4- vs. 6-weeks in our study), the method of swimming (bubbled stagnant water vs. the presence of strong currents in our study) or the strain of mice used (C57BL/6 vs. CD1 this study). In this regard, we find that C57BL/6 mice do not typically swim against water currents unlike CD1 mice (see Supplementary Video 1). We believe that swimming against water currents mimics more closely a physiological exercise stimulus and limits human intervention. Consistent with this, when C57BL/6 mice were exercised on a treadmill, the exercise-induced bradycardia was linked to an increased vagal and decreased sympathetic tone (De Angelis et al., 2004), as in our study. Clearly more studies are needed to uncover the source of these discrepancies.

Because we used pharmacological blockade to assess HR regulation by the ANS, it is important to consider the inevitable interactions between SNA and PNA (Taylor et al., 2001). Two-Way ANOVA revealed that SNA blockade lowered HR more in the presence of PNA blockade than without PNA blockade in all groups. These findings are predicted by “accentuated antagonism” mechanisms observed in animals (Brack et al., 2004) and humans (Uijtdehaage and Thayer, 2000), in which cardiac vagal activity inhibits the chronotropic effects of SNA through pre- (Löffelholz and Muscholl, 1969) and post-synaptic (Hartzell, 1988) mechanisms (Levy, 1971) which can occur via several mechanisms: (a) cardiac PNA withdrawal due to reduced blood pressure following SNA blockade (Billman, 2013), (b) inhibition of noradrenaline-release by cardiac sympathetic fibers by acetylcholine (Löffelholz and Muscholl, 1969), or (c) biochemical convergence of PNA and SNA on biochemical signaling in SA node cardiomyocytes (Hartzell, 1988; Brodde et al., 2001). The accentuated antagonist mechanism can also readily explain the ability of PNA blockade to cause 1.5- to 2-fold greater elevations in HR when given in the absence vs. the presence of SNA blockade.

Our study is also the first to comprehensively dissect the contributions of PNA vs. SNA on the magnitude and dynamics of HRV in exercised mice, providing support for the utility of HRV to non-invasively assess ANS regulation of HR in exercised mice. As in previous studies (Ishii et al., 1996; Aschar-Sobbi et al., 2015), the HRV spectrum was separated into HF (1–5 Hz) and LF (0.1–1 Hz) regions which differ from the ranges typically used in humans (0.04–0.15 and 0.15–0.4 Hz, respectively). Our results show that, unlike humans and larger mammals (Pagani et al., 1986; Goldsmith et al., 1992), most of the HRV in exercised and sedentary mice is associated with the HF region which is linked to blood pressure fluctuations associated with breathing (Akselrod et al., 1981). Moreover, the HF HRV in all mice is mediated primarily by PNA, consistent with previous studies in mice (Gehrmann et al., 2000; Thireau et al., 2008) as well as humans and dogs (1996, Motte et al., 2005; Billman, 2009). The conclusion that PNA controls the rapid HR fluctuations can be explained using the deBoer model (deBoer et al., 1987), by considering the rapid responses of muscarinic-dependent K^+^ channels (i.e., K, ACh) to changes in PNA (Olshansky et al., 2008).

We further found that LF HRV also depended on PNA in all groups of mice, similar to humans (Arai et al., 1989), while SNA blockade, either in the presence or absence of PNA blockade, had little effect on HRV, as reported in other mouse studies (Ishii et al., 1996; Gehrmann et al., 2000). The lack of dependence of LF HRV on SNA in mice has also been reported in humans (Kingwell et al., 1994; Moak et al., 2007; Rahman et al., 2011), even though LF HRV has been used in dogs and humans to assess SNA (Pagani et al., 1984, 1986). The basis for the discrepancies related to the role of SNA in LF HRV is unclear but may reflect complexities of the baroreceptor feedback gain (Pomeranz et al., 1985; Moak et al., 2007; Rahman et al., 2011) wherein HR regulation by SNA occurs with relatively large blood pressure perturbations as evoked with postural changes (Akselrod et al., 1981; Furlan et al., 2000) but not when subjects are maintained in supine positions (Pomeranz et al., 1985). Accordingly, mice may show limited blood pressure fluctuations under our experimental conditions thereby limiting the LF HRV (Eckberg, 1997). Alternatively, it is conceivable that the sympatholytic effects of isoflurane may reduce the SNA-dependent LF power in our mouse studies, as has been reported previously (Galletly et al., 1994). However, we found that the HR reductions and HRV elevations were similar in telemetry-implanted and anesthetized mice after 6 weeks of swim training (see Table 3). Future studies will clearly be needed to establish the basis for the differences between mice and men in the magnitude of LF HRV and the contributions of SNA.

**Table 3:** Heart rate and heart rate variability in ECG telemetry-implanted swim trained mice.

	**Baseline (*n* = 4)**	**6-week swim trained (*n* = 4)**	***P*-value**
HR (bpm)	506 ± 19	458 ± 20^*^	0.024
HF Power (s^2^)	4.5 ± 2.3	7.9 ± 2.8^*^	0.042

*HR, heart rate; HF power, high-frequency power. Data presented as mean ±SD*.

* *P < 0.05, compared with sedentary using Student's t-test. LF power could not be determined from ECG-telemetry data*.

It has been suggested that the high HRs associated with high SNA (Malik and Camm, 1993; Ishii et al., 1996) and low PNA can restrict the HRV in mice by creating a “ceiling effect” and thereby limiting HR responses to changes in autonomic activity. Additionally, increases in HR have been shown to limit HRV regardless of autonomic nerve activity (Gehrmann et al., 2000). Such mechanisms would potentially explain the elevated HRV seen in bradycardic exercised mice and are consistent with our observation that PNA blockade both elevated HR and reduced (abolished) HRV in sedentary and exercised mice, with much larger responses in the exercise groups. However, despite an exponential decay-like dependence of HRV on HR within each group of mice, as shown previously (Billman, 2013; Monfredi et al., 2014), the HR reductions produced in both sedentary and exercised mice following SNA blockade occurred without either notably elevating HRV or influencing the dependence of HRV on HR within each group. In addition, subsequent PNA blockade, after propranolol, markedly reduced HRV in the exercise mice despite increasing HR. Thus, in contrast to what has been argued in the literature (Monfredi et al., 2014; D'Souza et al., 2015), HR *per se* cannot be the sole (or primary) determinant of HRV, even though HRV correlated with HR within each group of mice (in the absence of PNA blockade), in agreement with one previous mouse study (Ishii et al., 1996) but not another (Gehrmann et al., 2000). Thus, since PNA blockade largely eliminated HRV, it seems reasonable to conclude that the dependence of HRV on HR is secondary to the level of PNA.

Despite the absence of a ceiling effect of HR on HRV in mice, we analyzed our HRV results with and without corrections for HR differences between the groups thereby allowing direct comparisons across studies and species (Billman, 2013; Sacha et al., 2013; Monfredi et al., 2014). Importantly, both types of exercise increased TP, LF, and HF HRV, with or without HR corrections. While increases in HR regulation by tonic PNA explains the enhanced HRV in our exercised mice, as predicted by the deBoer model (deBoer et al., 1987), it is important to appreciate that exercise has a multitude of other effects. For example, exercise causes profound vascular remodeling, which can directly affect the compliance properties of baroreceptors (Cameron and Dart, 1994; Kingwell et al., 1997) and the baroreflex feedback gain (Kirchheim, 1976; Brum et al., 2000), thereby explaining the benefits of exercise in cardiovascular disease patients (Iellamo et al., 2000). But exercise also attenuates the central gain of the baroreflex (Tipton et al., 1982; Chen et al., 1995) via remodeling of the paraventricular nucleus (PVN) as well as peripheral elements (Brum et al., 2000). Exercise has also been shown to enhance SA node responsiveness to vagal stimulation (Danson and Paterson, 2003; Mizuno et al., 2011) as well as modulate the levels of homeostatic factors such as oxytocin (Braga et al., 2000; Michelini, 2007). Clearly more studies will be needed to fully dissect the contribution of various tonic and reflex factors contributing to the bradycardia and increased HRV induced by exercise.

Interactions between PNA and SNA could also conceivably influence HRV. For example, in all groups we observed tendencies for HRV to increase following propranolol administration in the presence of atropine, which might reflect the indirect impact of HR on HRV. Indeed, when HR correction is applied, the effect of propranolol (in the presence of atropine) on HRV is slightly blunted. Thus, correcting the HRV using HR squared (Billman, 2013) may be insufficient to fully capture the independent effects of HR on HRV in mice which would also explain our inability to see vagally-mediated increases in either total or HF HRV following β-adrenergic blockade, as seen in humans (Kollai et al., 1994). Regardless, interactions between SNA and PNA may also be factors in limiting the impact of SNA on HRV in our mice by promoting or enhancing the effects of PNA, as reported previously in humans (Yang and Levy, 1984). These observations speak to the complex (and not simply opposing) interactions between the sympathetic and parasympathetic nervous systems and their modulation of HR.

### Limitations

Previous studies have concluded that isoflurane can inhibit both SNA (i.e., be sympatholytic) and PNA (Galletly et al., 1994), thereby limiting the amount of HRV (Kato et al., 1992; Galletly et al., 1994) and preventing direct comparisons with several previous studies using conscious mice (Ishii et al., 1996; Gehrmann et al., 2000; Guasch et al., 2013; D'Souza et al., 2014). However, we found no differences in the magnitude of HF power following swim training between anesthetized and conscious (telemetry-implanted) mice. Moreover, a distinct advantage of using anesthetized mice to assess HRV is that it provides the ability to control breathing rates, temperature, and movement which all influence HRV, thereby confounding the interpretation of HRV studies.

While our pharmacological and isolated atrial studies support the conclusion that PNA plays a dominant role in baseline HRV of sedentary mice as well as both the bradycardia and increased HRV induced by exercise, it would obviously be useful to measure cardiac sympathetic and vagus nerve activity and nodal tissue responsiveness to confirm these conclusions.

Our analysis of oxygen consumption (VO_2_) measurements was limited to the exercise period, allowing us to determine the “extra” work associated with exercise training and its impact on remodeling. In contrast to our exercise measurements, we did not have the ability to simultaneously measure VO_2_ and ambulatory activity during resting periods, precluding an estimate of total O_2_ consumption over the training period. It is possible that mice that were subjected to forced swim exercise may be less active during the resting period compared to free wheel mice, given the greater intensity of the exercise and the potential impact of sleep interruption on ambulatory activity.

We did not simultaneously measure blood pressure which limits our ability to correct for potential baroreceptor-induced changes in HR responses following ANS blockade.

## Conclusion

Our studies establish for the first time that both forced swim and voluntary free-wheel endurance training decreased HR and increased HRV, as seen in the hearts of athletes. Moreover, these HR and HRV changes with exercise are linked primarily to enhanced HR regulation by the PNA. Our findings support the utility of both forced and voluntary exercise to recapitulate the athlete's heart phenotype and suggest HRV can be a useful tool for assessing changes in dynamic HR modulation by the ANS in mouse models of health and disease.

## Author contributions

Experiments were performed at the University of Toronto Department of Physiology and York University Department of Biology, respectively. The conception and design of the work were carried out by all authors. Acquisition, analysis and interpretation of the Data were performed by RL, CG, FI, and PB. All authors contributed in drafting or critically revising the manuscript. All authors approved the final version of this manuscript and agreed to be accountable for all aspects of the work. All persons designated as authors qualify for authorship and all those who qualify for authorship are listed.

### Conflict of interest statement

The authors declare that the research was conducted in the absence of any commercial or financial relationships that could be construed as a potential conflict of interest.

## Abbreviations

ANS: Autonomic nervous system
BW: Body weight
CO: Cardiac output
EF: Ejection fraction
FFT: Fast Fourier Transform
FS: Fractional shortening
HR: Heart rate
HRV: Heart rate variability
HF: High frequency
IBI: Inter-beat-interval
LVDd: Left ventricular diastolic diameter
LVDs: Left ventricular systolic diameter
LVPWth: Left ventricular posterior wall thickness
LF: Low frequency
PNA: Parasympathetic nerve activity
SA: Sinoatrial
SNA: Sympathetic nerve activity
TP: Total power.
